# Barrett’s esophagus: review of diagnosis and treatment

**DOI:** 10.1093/gastro/got015

**Published:** 2013-04-30

**Authors:** Raja Shekhar Sappati Biyyani, Amithab Chak

**Affiliations:** Digestive Health Center, University Hospitals Case Medical Center, 11100 Euclid Ave, Cleveland, OH 44106, USA

**Keywords:** Barrett’s esophagus, endoscopic mucosal resection, endoscopic submucosal dissection

## Abstract

Barrett's esophagus (BE) is an acquired condition characterized by replacement of stratified squamous epithelium by a cancer predisposing metaplastic columnar epithelium. Endoscopy with systemic biopsy protocols plays a vital role in diagnosis. Technological advancements in dysplasia detection improves outcomes in surveillance and treatment of patients with BE and dysplasia. These advances in endoscopic technology radically changed the treatment for dysplastic BE and early cancer from being surgical to organ-sparing endoscopic therapy. A multimodal treatment approach combining endoscopic resection of visible and/or raised lesions with ablation techniques for flat BE mucosa, followed by long-term surveillance improves the outcomes of BE. Safe and effective endoscopic treatment can be either tissue acquiring as in endoscopic mucosal resection and endoscopic submucosal dissection or tissue ablative as with photodynamic therapy, radiofrequency ablation and cryotherapy. Debatable issues such as durability of response, recognition and management of sub-squamous BE and optimal management strategy in patients with low-grade dysplasia and non-dysplastic BE need to be studied further. Development of safer wide field resection techniques, which would effectively remove all BE and obviate the need for long-term surveillance, is another research goal. Shared decision making between the patient and physician is important while considering treatment for dysplasia in BE.

## INTRODUCTION

In response to injury associated with gastroesophageal reflux, the normal stratified squamous epithelium of the esophagus may be replaced by a metaplastic columnar intestinal-like epithelium—Barrett’s esophagus (BE)—which is predisposed to cancer development [1]. Three types of Barrett’s columnar epithelia have been described—a junctional (cardia) type-, a gastric fundic type- and intestinal-type metaplasia, the latter being specialized columnar epithelium, with prominent goblet cells [2]. Barrett’s epithelium appears to progress sequentially from intestinal metaplasia (IM) to low-grade dysplasia (LGD) to high-grade dysplasia (HGD) and finally to invasive adenocarcinoma. Although BE is associated with a low (0.5%) annual incidence of HGD or esophageal adenocarcinoma, a four-fold increase in incidence of esophageal cancer has been noted in certain patient populations [3, 4, 5]. Five-year survival with esophageal adenocarcinoma remains a dismal 13–16% [6, 7].

Since the description of BE 50 years ago, there have been tremendous advances in understanding the biology of BE, risk factors and progression towards cancer, and enhanced endoscopic imaging techniques for identification of dysplasia within BE. Despite the high mortality and morbidity associated with surgical resection, esophagectomy was once considered the therapeutic ‘gold standard’ for BE with HGD, due to a concern over a high risk of harboring occult invasive cancer [8–13].

The management of BE with dysplasia and early cancer has changed radically from morbid surgical resection to organ-sparing endoscopic therapy. With the advent of a multitude of safe and effective treatment options—such as endoscopic mucosal resection (EMR) and endoscopic sub-mucosal dissection (ESD) in combination with tissue ablative therapies, such as photodynamic therapy (PDT), radiofrequency ablation (RFA) and cryotherapy—endoscopic therapy has become the standard of care in expert centers throughout the world.

### Endoscopic diagnosis of BE

BE is both an endoscopic and pathologic diagnosis. Endoscopic knowledge of the anatomy of the gastro-esophageal junction (GEJ) is key in the diagnosis of BE. During endoscopy, after gastric decompression, the endoscope should be withdrawn slowly to identify the diaphragmatic hiatus, the top of the gastric folds and the squamo-columnar junction (SCJ or Z-line) which coincide and are normally at the same distance from the incisors. BE is endoscopically suspected when the SCJ is proximal to the top of the gastric folds, with the presence of salmon-colored mucosa within this distance. Endoscopic biopsies should be taken from within this area to confirm the diagnosis of BE [14]. BE is classified as (i) short-segment BE (SSBE) when the distance between the top of the gastric folds and the SCJ is less than 3 cm and (ii) long-segment BE (LSBE), when the distance is greater than 3 cm. The Prague C and M criteria represent the standard classification system and categorize BE more precisely, based on the circumferential extent (C) and the maximum extent (M) of Barrett’s metaplasia [15]. Documenting the length of the BE has prognostic implications and may influence the method of ablation in the event of HGD or IMC being found.

### Endoscopic screening of BE

American Gastroenterological Association (AGA) recommendations for screening for BE are shown in Table 1 [1]. The current practice of screening for BE with EGD in the general population with gastroesophageal reflux disease (GERD) is controversial and should be considered on a case-by-case basis. Traditionally, endoscopic screening for BE has been reserved for male Caucasians with long-term GERD. However, BE is known to be present in patients without GERD and up to 57% of patients with esophageal adenocarcinoma never report symptoms of typical GERD, limiting this approach and missing a significant portion of patients at risk for esophageal adenocarcinoma [16–19]. Similarly, the population prevalence of BE is 2–7% and the risk of HGD or IMC is only about 0.5% per year, making population screening a strategy that is not very cost-effective [3, 20, 21]. With the advent of newer and cheaper approaches for diagnosis of BE—such as unsedated examinations and non-endoscopic options (capsule esophagoscopy and cytosponge)—the cost-effectiveness of screening may improve [22–24].

**Table 1:** AGA recommendations for screening for Barrett’s esophagus

*Screen patients with multiple risk factors associated with esophageal adenocarcinoma:
•Age 50 years or older •Male gender •White race •Chronic GERD •Hiatal hernia •Elevated body mass index (BMI) •Intra-abdominal distribution of body fat
*Recommend against screening the general population with GERD

### Endoscopic surveillance of BE

AGA guidelines on endoscopic surveillance of BE are as shown in Table 2 [1]. Although the special image-enhanced endoscopic technique is not usually required, a high-resolution endoscope (>850 000 pixels) should be used to evaluate patients with BE and standard-resolution endoscopes are not recommended [25]. Currently, endoscopic surveillance is suggested for patients without BE-related dysplasia and in patients with LGD in BE not opting for ablation. In contrast, surveillance without therapy for HGD is highly controversial and no longer practiced by most clinicians. Even after ablation for dysplastic BE, surveillance is performed based on the highest degree of dysplasia prior to ablation. In contrast to endoscopic surveillance practice in North America, the British societies and other groups do not require IM and survey all columnar epithelium in the esophagus. AGA endoscopic surveillance recommendations include detailed endoscopic evaluation using white light endoscopy, followed by biopsy specimens of any mucosal irregularities and four-quadrant biopsy specimens obtained at least every 2 cm. If dysplasia is suspected, then the four-quadrant biopsy specimens should be obtained every 1 cm [1].

**Table 2:** Professional society guidelines for surveillance intervals

Organization	Surveillance interval		
	No dysplasia	LGD	HGD
**American College of Gastroenterology (ACG)**	•Two EGD’s with biopsy in first year •EGD every 3 years if no dysplasia	•Repeat in 6 months. •Then yearly until no dysplasia x 2	•Expert pathologist confirmation •EMR for mucosal irregularity •Definitive treatment or Surveillance every 3 months
**American Gastroenterological Association (AGA)**	•Two EGD’s with biopsy in first year •EGD every 3–5 years if no dysplasia	•Every 6–12 months	•Consider definitive treatment. •If not, EGD every 3 months
**American Society for Gastrointestinal Endoscopy (ASGE)**	•Two EGD’s with biopsy in first year •EGD every 3 years if no dysplasia	•Every 6 months x 2 •Then yearly	•Consider definitive treatment. •If not, EGD every 3 months for a year with large caliber forceps.
**British Society of Gastroenterology (BSGE)**	•Every two years	•Acid suppression for 8–12 weeks followed by repeat EGD. •EGD every 6 months if LGD persists •EGD every 2–3 years if no dysplasia x 2	•Intervention OR •EGD every 6 months

The interpretation of dysplasia can be a matter of contention. At least two experienced gastrointestinal pathologists should evaluate all Barrett’s biopsies when a diagnosis of dysplasia is considered [25]. The use of large-capacity or ‘jumbo’ forceps may improve tissue acquisition and dysplasia detection [26]. Rigorous surveillance with a systematic biopsy protocol improves detection of dysplasia and early cancers [27]. In addition, patients with BE in a surveillance program may have cancers that are detected at an earlier stage, with improved survival [28, 29]. Narrow-band imaging (NBI), chromo-endoscopy, optical coherence tomography, confocal-microendoscopy, spectroscopic probe and endoscopic image enhancement technology (such as ‘i-scan’) may be helpful for targeting biopsies during surveillance of BE for dysplasia but, in large part, these novel imaging technologies remain experimental [30–38].

### Endoscopic treatment of BE

The key rationale of endoscopic treatment is to resect and/or ablate the dysplastic mucosa, followed by acid suppression to permit re-epithelialization with neosquamous mucosa. Patients with HGD are at high risk for recurrence and it is thus important to ablate the residual metaplastic epithelium after the dysplastic epithelium has been addressed [39–44]. By eradicating dysplasia and intestinal metaplasia (IM), the cancer rate may decrease, leading to improved survival [39–44].

Accurate pre-treatment staging is essential to ensure an appropriate choice of therapy and optimal long-term outcomes. An accepted multimodal endoscopic treatment approach is targeted EMR of visible lesions, in combination with one or more ablative therapies after a confirmed BE pathology report. Endoscopic treatment can be tissue-acquiring, as in endoscopic mucosal resection (EMR), and endoscopic sub-mucosal dissection (ESD) or tissue ablative, as with photodynamic therapy (PDT), radiofrequency ablation (RFA) and cryotherapy. Treatment is then tailored after detailed discussion of the available endoscopic treatment options including risks, benefits and surveillance as an alternative.

HGD has a higher risk of concomitant cancer and a 6% per year rate of progression to cancer [45–47]. A greater emphasis on accurate diagnosis of BE with HGD, as well as better prediction of risk for progression to esophageal adenocarcinoma (EAC), has been advocated [45–47, 50]. Hence treatment of dysplastic BE is now widely acknowledged and preferred over surveillance [45–50]. However, recent studies confirmed a much smaller risk of occult cancer with HGD and <1% incidence of lymph node metastasis with intra-mucosal cancer (IMC) [11–13, 49]. Endoscopic therapy for BE with HGD is highly effective, safe, with a long-term survival rate similar to esophagectomy [42–44]. In patients with multifocal HGD, the risk of occult cancer is higher and selected patients may be considered for surgery [45–51].

Similarly to HGD, the long-term survival rate of patients with BE and intra-mucosal cancer (IMC) undergoing endoscopic therapy is equal to patients undergoing surgery [42–44]. Extensive EMR for removal of BE with early neoplasia is thought to be safe, with no procedure-related perforations or mortality, but strictures have been reported in 27% and major bleeding in 2% [48]. Outcomes for complete BE eradication are modest at 49.4% and eradication of high-grade dysplasia at 81%. Barrett's length of less than 5 cm is the only significant predictor of complete response [48]. Dunbar *et al.* reported a 1–2% risk of unexpected lymph-node metastases in patients with BE and IMC [49]. EMR and less so endoscopic ultrasound (EUS) in non-nodular BE helps with diagnosis of sub-mucosal invasion, which is associated with a higher nodal metastasis risk and requires surgery or systemic therapy [13, 49–51]

Management of low-grade dysplasia (LGD) is somewhat controversial. High inter-observer variability among the pathologists in diagnosis LGD seems to affect the natural history of LGD and its rate of progression to HGD and cancer [52]. High rates of eradication of intestinal metaplasia (IM) and LGD, using RFA as reported, is enticing [54]. However, the survival benefits and cost-effectiveness of ablation over surveillance are not clear as estimated from a modeling study [55]. This study estimated the risk of progression rate of 0.7% per year and concluded that although patients with LGD can be managed optimally with ablation, long-term post-ablation surveillance may not be cost-effective [55]. At this time, offering ablation to patients with LGD is made on a case-by-case base and the decision is a shared one between the physician and the patient. Young age at diagnosis, presence of multifocal LGD and LGD on several biopsy sessions may pose a higher risk of progression and, hence, are candidates for ablation [55].

Even though RFA can eradicate 92% of non-dysplastic Barrett’s esophagus (NDBE) with relatively low complication rate and a durable response, the absolute rate of progression to cancer in these patients is low and routine ablation of NDBE is not currently recommended. Histological changes in the gastric cardia, with development of nodules, dysplasia and adenocarcinoma after ablation of BE, have been reported and this calls for caution while considering ablation of BE with LGD or NDBE [56–58].

### Mucosal resection

The goal of endoscopic treatment is resection of the mucosa and sub-mucosa of the targeted area to the *lamina propria*. Endoscopic treatment is not only curative but also allows for histological assessment of the resected specimen, which helps to accurately stage the lesion by assessing the depth of the tumor, involvement of lateral and deep margins, lymphatic and vascular invasion [59–69]. EMR and ESD are two organ-sparing endoscopic treatment techniques developed for removing tumors limited to the mucosa—and occasionally sub-mucosa—in the esophagus and elsewhere in the GI tract.

**Figure 1:**
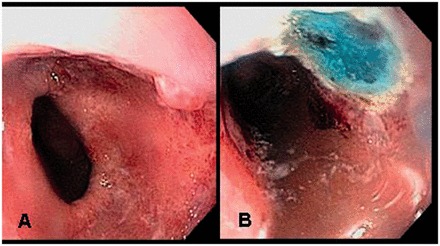
EMR of Barrett’s HGD nodular lesion. A: Nodular lesion within the Barrett’s mucosa. B: Post-EMR image.

Inoue *et al.* were the first to describe the use of EMR for early gastrointestinal cancers, including esophageal cancer [59] (Fig. 1)*.* EMR can be injection-, cap- or ligation-assisted. EMR can be performed *en bloc* for smaller lesions (<2 cm) or piecemeal [59–67]. Most endoscopists are familiar with band ligation and this technique has gained in popularity. The two techniques appear similar in terms of the depth of resection, efficacy and safety [59–67]. Although, in some situations, the cap technique may yield slightly larger pieces, the band ligation assisted method saves cost and time [59–67].

EMR leads to complete remission rates of 97–100% with 5-year survival rates of 84–98% and 21.5% rate of recurrence with metachronous lesions [59–67]. Ablative therapy after ER could decrease this risk [68–70]. Complications of EMR include bleeding, stricture formation and stenosis. Mucosal defects involving over three-fourths the circumference of the esophagus and mucosal defects longer than 30 mm are associated with greater severity of stenosis [59–72]. Complete Barrett’s eradication EMR (CBE-EMR) with a reported 97.5% efficacy is a recently introduced concept, wherein the entire length of BE is eradicated in multiple sessions [72]. CBE-EMR also provides for the most accurate staging of BE with neoplasia, at a cost of a high rate of esophageal stenosis (49.7%) [72]. In a European, multicenter, randomized study of 43 patients, the efficacy of CBE-EMR was similar to RFA for eradication of all IM (96 vs 95%), but was associated with much higher rates of bleeding (23 vs 5%) and stricture formation (86 vs 14%) [72].

**Figure 2:**
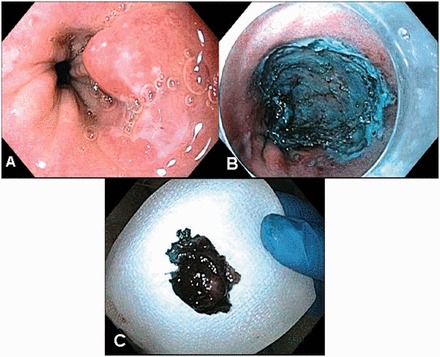
ESD of Barrett’s HGD nodular lesion. A: Nodular lesion within the Barrett’s mucosa. B: Post-ESD image. C: Gross specimen.

Endoscopic sub-mucosal dissection (ESD) has been developed for *en bloc* resection and removal of larger than 2 cm flat GI tract lesions [73–78] (Fig. 2). Feasibility of ESD for early esophageal cancers has been demonstrated in small case series from Asia and Europe [74–78]. Even though ESD may have a better rate of tumor-free margins for resection, it is technically challenging, associated with complications such as perforation and stricture formation [74–78]. At the present time, there is no evidence to suggest that ESD is superior to EMR with ablation to achieve complete remission and improve survival in patients with BE and early cancer (73).

### Mucosal ablation

Mucosal ablation using endoscopic laser therapy, such as multi-polar electro coagulation (MPEC) and argon plasma coagulation (APC), was demonstrated almost two decades ago [79–82]. Photodynamic therapy (PDT) was a relatively new therapy. However, low response rates and high rates of adverse reactions, such as strictures and risk of buried Barrett’s glands, led to replacement of these above techniques with radiofrequency ablation (RFA) and cryotherapy.

Photodynamic therapy **(**PDT) utilizes the photochemical energy of a photosensitizer [porphimer sodium (Ps), 5-aminolevulinic acid (5-ALA) or m-tetrahydroxyphenylchlorin (mTHPC)], which is concentrated in neoplastic tissue, followed by activation with endoscopically delivered laser light (balloon based or bare cylinder) of an appropriate power and wavelength. The activated drug reacts with oxygen, generating free radicals, and induces cell membrane damage and apoptosis [83]. The greatest body of data pertaining to efficacy and long-term outcomes in the treatment of BE with dysplasia or IMC is related to porphimer sodium [84]. PDT (using Ps) with PPI was more effective than PPI alone in eradicating BE with HGD (77% vs 39%), along with a lower rate of progression to cancer (13% vs 28%) and a significantly longer time to progression [84]. Only 48% of patients with PDT remained in complete remission, compared to 4% of those on PPI alone [84].

Photosensitivity (69%), esophageal strictures (36%), chest pain (20%), fever (20%) and dysphagia (19%) are the common side-effects of PDT [84–91]. Older age, smoking and presence of residual non-dysplastic BE may result in recurrence and/or presence of buried Barrett’s glands [84–91]. Adenocarcinoma can arise from buried Barrett’s glands and limit the effectiveness of PDT therapy [84–91].

**Figure 3:**
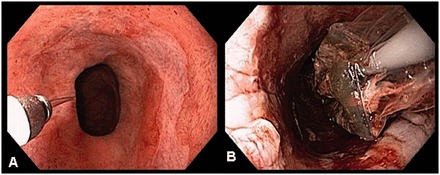
RFA of Barrett’s HGD flat mucosa. A: Barrett’s mucosa pre-RFA. B: Barrett’s mucosa with tissue coagulum post-RFA.

Radiofrequency ablation (RFA) uses an alternating electrical current to induce an electromagnetic field [92–95]. The electromagnetic field causes charged ions to rapidly oscillate, collide with one another and create molecular friction and a rapid, exothermic release of thermal energy, resulting in controlled thermal injury [92–95]. The coagulated mucosal tissue acts as an insulator, limiting the ablation depth in a superficial, controlled and consistent manner (Fig. 3). There are two commercially available devices to perform RFA in the esophagus: the HALO360 and HALO90 (BARRX Medical, Inc, Sunnyvale, CA, USA).

In a prospective, multicenter study of dysplastic BE patients, complete remission of IM (CRIM) was seen in 77% and complete remission of dysplasia (CRD) was seen in 86% [92–95]. In patients with HGD, CRIM was seen in 74% and CRD in 81%. There was less disease progression (3.6%) and fewer cancers noted (1.2%) in patients from the ablation group and the response sustained for 2–3 years [92–95]. However, more long-term studies are needed to demonstrate continued durability.

Non-cardiac chest pain (8.9%), nausea (7.5%), bleeding (1.6%) and minor discomfort requiring pain medications (44%) are the common complications of RFA [92–95]. Serious complications seen with RFA, such as strictures (6.4%), buried Barrett’s and dysplasia (0.5–1%), are much less than the complications observed with PDT [92–95]. Given the superficial nature of thermal injury and requirement of adequate tissue apposition, RFA may not be appropriate in patients with nodular BE. However, RFA can be successfully performed after focal EMR of nodular or visible lesion. Akiyama *et al.* retrospectively studied RFA outcomes in Barrett’s patients with acid suppression [96]. Despite treatment with proton-pump inhibitors (PPI), about 29% of the 45 patients treated with RFA for BE still exhibited moderate-to-severe esophageal acid exposure (EAE) [96]. Increased reduction of BE surface area and complete eradication of BE were noted in patients with normal–mild-, compared to moderate–severe, EAE (99 vs 95%) [96]. RFA is currently the best available ablation technique for treatment of flat HGD and for eradication of residual BE mucosa after focal EMR [25].

**Figure 4:**
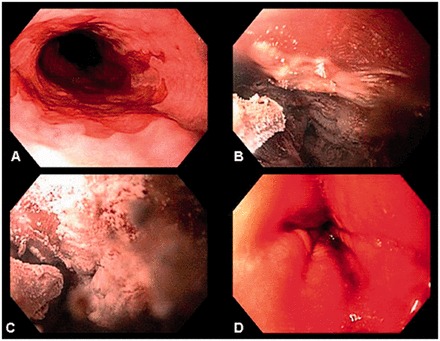
Cryotherapy of Barrett’s HGD flat mucosa. A: Barrett’s mucosa pre-cryotherapy. B & C: Cryotherapy catheter application with cycles of rapid freezing. D: Thawing cycle slow redness indicating direct cell injury.

Pasricha *et al.* described the use of endoscopic cryotherapy, wherein application of a cryogen (liquid CO_2_ or liquid N_2_) to the BE, with repeated cycles of rapid freezing and slow thawing, causes direct cell injury, vascular stasis and cellular apoptosis [97–104]. The efficacy depends on the tissue temperature, duration of freezing, cooling rate, thaw rate, number of freeze–thaw cycles and interval between the cycles [97–104] (Fig. 4).

EMR for resection of visible lesions, followed by cryotherapy, was prospectively studied in non-surgical BE patients with HGD. The treatment resulted in 90% improved histology and 30–40% complete resolution of dysplasia [97–104]. Similarly 97% HGD eradication and 57% IM eradication was noted in a multicenter, retrospective cohort study of 98 patients with BE and HGD at a 10.4 month follow-up following a mean total of four treatments [97–104]. Management of local disease in unresectable cancer is another application of endoscopic cryotherapy. In a retrospective study of 79 subjects with T1–T4 cancer and a mean tumor burden of 4 cm, complete response of intraluminal disease was seen in 61% (75% with mucosal cancer), with 13% developing benign strictures [102].

Common side-effects include strictures (3%) and chest pain (2%). Buried Barrett’s was seen in 3% [97–104]. Contra-indications to esophageal cryotherapy ablation include mucosal breaks, coagulopathy and retained food in the stomach [97–104]. Altered surgical anatomy, eosinophilic esophagitis and presence of large hiatal hernia pose significant risk of perforation, due to restricted volume or distensibility of the gastrointestinal tract [97–104]. Similarly to RFA, cryotherapy appears promising, with good efficacy and safety profile. However larger studies and long-term durability data of treatment are necessary.

## CONCLUSION

Endoscopic therapy in an appropriately selected patient population appears to be safe and effective for management of BE with dysplasia and IMC. BE eradication is recommended for treatment and prevention of metachronous and synchronous lesions. Further studies are needed to assess the long-term durability of endoscopic therapy, to recognize and manage buried Barrett’s and to identify optimal management strategy in patients with LGD and non-dysplastic BE. Management of BE is a dynamic process and will continue to evolve as we make advances in our understanding of the development of dysplasia and cancer in BE, genetics of BE, identify molecular markers or less-expensive methods of screening and surveillance for cancer and dysplasia and develop safer wide-field resection techniques, which would effectively remove all Barrett’s and obviate the need for long-term surveillance.

BE with dysplasia and cancer often entails complex decision-making. Its management requires a multidisciplinary approach, in collaboration with expert endoscopists, surgeons, oncologists and pathologists. A clear understanding of the biology of BE—risk of neoplastic progression, appropriate screening and surveillance, patient selection, availability of various endoscopic ablation techniques, their benefits, risk profile and applicability to the patient to be treated—will help successful endoscopic and/or surgical management of BE.

**Conflict of interest:** none declared.
